# Dickkopf-1 Inhibition Reactivates Wnt/β-Catenin Signaling in Rhabdomyosarcoma, Induces Myogenic Markers In Vitro and Impairs Tumor Cell Survival In Vivo

**DOI:** 10.3390/ijms222312921

**Published:** 2021-11-29

**Authors:** Irina Giralt, Gabriel Gallo-Oller, Natalia Navarro, Patricia Zarzosa, Guillem Pons, Ainara Magdaleno, Miguel F. Segura, Constantino Sábado, Raquel Hladun, Diego Arango, José Sánchez de Toledo, Lucas Moreno, Soledad Gallego, Josep Roma

**Affiliations:** 1 Laboratory of Translational Research in Child and Adolescent Cancer, Vall d’Hebron Research Institute, Hospital Universitari Vall d’Hebron, Universitat Autònoma de Barcelona, 08035 Barcelona, Spain; giralt.irina@gmail.com (I.G.); gabriel.gallo@vhir.org (G.G.-O.); natalia.navarro@vhir.org (N.N.); patricia.zarzosa@vhir.org (P.Z.); guillem.pons@vhir.org (G.P.); ainara.magdaleno@vhir.org (A.M.); miguel.segura@vhir.org (M.F.S.); jossanchez@vhebron.net (J.S.d.T.); lucas.moreno@vhebron.net (L.M.); 2 Pediatric Oncology and Hematology Department, Hospital Universitari Vall d’Hebron, Universitat Autònoma de Barcelona, 08035 Barcelona, Spain; csabado@vhebron.net (C.S.); rhladun@vhebron.net (R.H.); 3 Group of Molecular Oncology, IRB Lleida, 25198 Lleida, Spain; darango@irblleida.cat

**Keywords:** Wnt antagonists, Dickkopf proteins, DKK, β-catenin, Wnt pathway, rhabdomyosarcoma, differentiation

## Abstract

The Wnt/β-catenin signaling pathway plays a pivotal role during embryogenesis and its deregulation is a key mechanism in the origin and progression of several tumors. Wnt antagonists have been described as key modulators of Wnt/β-catenin signaling in cancer, with Dickkopf-1 (DKK-1) being the most studied member of the DKK family. Although the therapeutic potential of DKK-1 inhibition has been evaluated in several diseases and malignancies, little is known in pediatric tumors. Only a few works have studied the genetic inhibition and function of DKK-1 in rhabdomyosarcoma. Here, for the first time, we report the analysis of the therapeutic potential of DKK-1 pharmaceutical inhibition in rhabdomyosarcoma, the most common soft tissue sarcoma in children. We performed DKK-1 inhibition via shRNA technology and via the chemical inhibitor WAY-2626211. Its inhibition led to β-catenin activation and the modulation of focal adhesion kinase (FAK), with positive effects on in vitro expression of myogenic markers and a reduction in proliferation and invasion. In addition, WAY-262611 was able to impair survival of tumor cells in vivo. Therefore, DKK-1 could constitute a molecular target, which could lead to novel therapeutic strategies in RMS, especially in those patients with high DKK-1 expression.

## 1. Introduction

The Wnt/β-catenin signaling pathway plays a pivotal role during embryogenesis and its deregulation is a key mechanism in many tumors [1]. Among the elements that act in its molecular deregulation, Wnt antagonists have been described as key modulators of Wnt signaling in cancer. Several families of Wnt antagonists have been described to date, and the Dickkopf (DKK) family stand out for being one of the most studied, and for having oncogenic involvement. DKK are soluble proteins that interact with the low-density lipoprotein receptor-related protein (LRP) 5/6 receptor, avoiding the formation of the Fz-LRP6 complex. In humans, four DKK genes have been identified: *DKK-1*, *DKK-2*, *DKK-3* and *DKK-4*, with *DKK-1* being the most studied and characterized [2,3].

In recent years, the therapeutic potential of targeting DKK-1 has been evaluated for several diseases, such as rheumatoid arthritis [4]. Although a dual role in some cancers has been described for DKK-1 [5,6,7], its oncogenic function is predominant and supports its inhibition as a therapeutic approach. The compound WAY-262611 was the first small molecule reported as able to inhibit DKK-1. The inhibition exerted by this compound facilitates Wnt3a-LRP5 interaction and blocks the formation of the DKK-1-LRP5-Kremen complex, thereby enabling activation of the Wnt/β-catenin pathway [5]. Although the effects of DKK-1 pharmacological inhibition have not been evaluated in cancer, it has been shown that WAY-262611 inhibits both cell migration and expression of focal adhesion kinase (FAK) in fibroblast-like synoviocytes [4].

Despite the fact that DKK-1 has been studied in several cancers, little is known about its function in pediatric tumors. Thus, only a few works have explored DKK-1 implication in medulloblastoma [6], neuroblastoma [7] and osteosarcoma [8]. In rhabdomyosarcoma (RMS), it has been shown that a reduction of DKK-1 expression results in myogenic differentiation in vitro [9]. Within pediatric tumors, RMS is the most common soft tissue sarcoma, accounting for around 5% of childhood cancers [10]. RMS is traditionally divided into two major subtypes based on its histological features: (1) embryonal RMS (ERMS) is the most common subtype (70–80% of cases); and (2) alveolar RMS (ARMS), representing 20–30% of cases, is the last subtype and the most aggressive one [11]. Despite the application of the most effective multimodal therapies, the survival rate for RMS remains around 65%. In addition, RMS patients with metastatic disease at diagnosis have a survival rate below 40%, despite intense treatments [12]. This particular group of patients needs new therapeutic approaches, mainly those who still do not respond adequately to current treatment protocols [13].

Herein, for the first time, we analyzed the therapeutic potential of DKK-1 pharmacological inhibition in RMS. Thus, after genetic or pharmacological inhibition in RMS cell lines, our results indicated that DKK-1 inhibition affects differentiation with after effects on proliferation and invasiveness. Furthermore, the compound WAY-262611 was able to impair the survival of tumor cells after tail-vein injection of RMS cells in an in vivo murine experimental model. Taken together, our results highlight DKK-1 as an interesting target that could lead to novel therapeutic strategies for RMS.

## 2. Results

### 2.1. Analysis of DKK Expression in RMS

**Figure 1:**
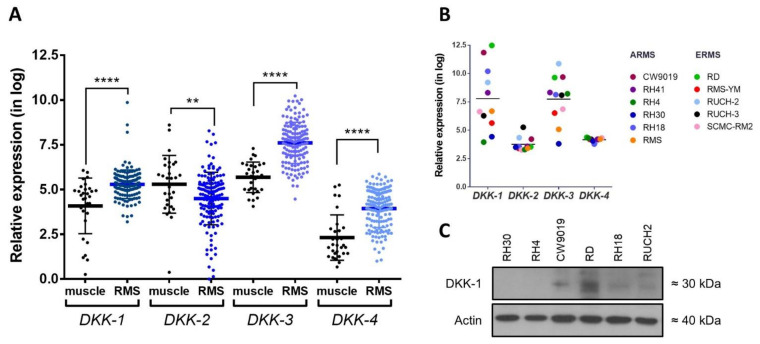
Expression analysis of DKK family members in RMS. (**A**) Relative mRNA expression of *DKK* genes in RMS patients, extracted from expression datasets, compared with muscle as the matched healthy counterpart. (**B**) Analysis of expression dataset revealed remarkable *DKK-1* and *DKK-3* overexpression in RMS cell lines. (**C**) Representative Western blot images of DKK-1 protein expression in a panel of RMS cell lines. ** *p* < 0.01 and **** *p* < 0.0001.

Since little is known about the therapeutic potential of DKKs in RMS, our first step was to investigate the expression levels of the four DKK members in RMS gene expression datasets (Figure 1A). The relative levels of *DKK-1*, *DKK-3* and *DKK-4* were significantly increased in RMS patients compared with muscle tissue from healthy donors. No differences between the ERMS and ARMS subtypes were observed (data not shown). However, the most prominent overexpression was observed for *DKK-1* and *DKK-3*. This last observation was also confirmed when DKK expression was analyzed in a set of RMS cell lines (Figure 1B) [14]. In the dataset analyzed, RD and CW9019 cell lines were those with the highest level of *DKK-1* expression. At the protein level, both cell lines also displayed the highest level of DKK-1 (Figure 1C).

### 2.2. Genetic DKK-1 Inhibition Led to Reduced Proliferation in RMS Cells

**Figure 2:**
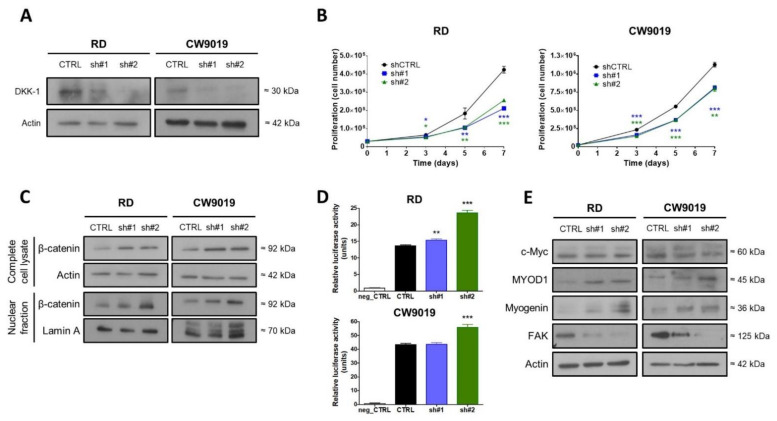
Analysis of DKK-1 genetic inhibition by shRNA. (**A**) Two shRNAs against DKK-1 (sh#1 and sh#2) were selected once the inhibition at the protein level was confirmed in two RMS cell lines. (**B**) The proliferation assay was carried out for 7 days in RD and CW9019 cell lines. (**C**) Activation of the Wnt signaling pathway after DKK-1 inhibition was analyzed by determination of β-catenin protein levels by WB in complete cell lysate and nuclear fraction, and by (**D**) β-catenin promoter activity using the TOPFlash system. In (**E**), the protein level of different specific markers was determined by WB. * *p* < 0.05, ** *p* < 0.01 and *** *p* < 0.001.

Despite the therapeutic potential of targeting DKK family members, only DKK-1 inhibitors have been developed to date. For this reason, we focused our efforts on DKK-1 and, consequently, we performed our DKK-1 genetic inhibition experiments in the two cell lines with the highest DKK-1 expression levels: RD (ERMS subtype) and CW9019 (ARMS subtype). Two shRNAs (sh#1 and sh#2) induced a clear DKK-1 downregulation in both cell lines (Figure 2A). Concomitantly, shRNA-mediated DKK-1 downregulation promoted a cell proliferation decrease in both cell lines (Figure 2B).

### 2.3. DKK-1 Knockdown Activated Wnt Signaling and Induced Expression of Myogenic Markers

In order to investigate the Wnt signaling status after DKK-1 knockdown, β-catenin levels in total cell lysate and in the nuclear fraction were analyzed by Western blot. Levels of β-catenin displayed an increase in both fractions and in both cell lines in response to DKK-1 silencing. The increase observed was particularly significant for nuclear β-catenin and with a particularly intense upregulation with sh#2 (Figure 2C). In addition, after DKK-1 genetic inhibition, an increase in β-catenin promoter activity was observed in both cell lines with sh#2. In cell line RD, a weak increase was also observed for sh#1 (Figure 2D). Levels of c-Myc protein were assessed as a canonical downstream target gene of Wnt signaling. Despite the increase of β-catenin at the protein level and the activation of its promoter activity, levels of c-Myc were unaltered in both cell lines (Figure 2E). Conversely, DKK-1 inhibition effected an increase in myogenic markers, such as MYOD1 and myogenin (Figure 2E). In addition, FAK levels, which are a direct regulator of cell adhesion and invasion, were strongly reduced in both RMS cell lines after DKK-1 knockdown (Figure 2E).

### 2.4. DKK-1 Pharmacological Inhibition Decreased Cell Proliferation and Invasion Rates

**Figure 3:**
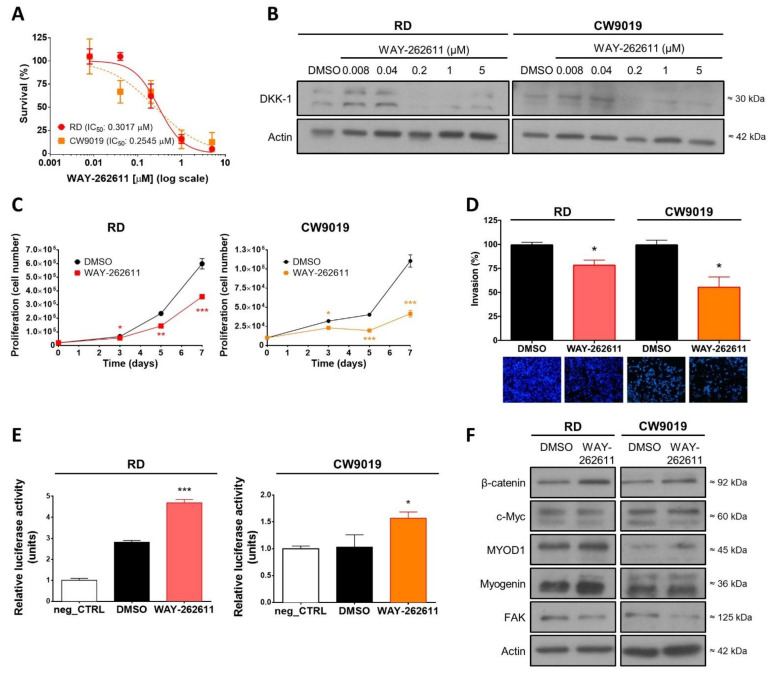
Pharmacological inhibition of DKK-1 by the compound WAY-262611. WAY-262611 treatment was carried out in both RMS cell lines (RD and CW9019). (**A**) The IC50 determination was assessed by a crystal violet proliferation assay after 72 h of WAY-262611 treatment. (**B**) DKK-1 protein expression after treatment with increasing concentrations of WAY-26211 was assessed by Western blot. (**C**) Proliferation over 7 days and (**D**) invasion analysis were carried out in both cell lines in presence of WAY-262611 at 0.2 µM. (**E**) β-catenin promoter activity was assessed by luciferase assay. (**F**) β-catenin levels, and those of other protein markers, were assessed by Western blot after treatment with WAY-262611 (0.2 µM). * *p* < 0.05, ** *p* < 0.01 and *** *p* < 0.001.

The results of our analysis revealed that the RD and CW9019 cell lines were both sensitive to WAY-262611 treatment, with IC50 values as low as 0.30 μM and 0.25 μM, respectively (Figure 3A). Despite the fact that the molecular mechanism for WAY-262611 is based on the inhibition of Kremen-LRP5/6-DKK-1 complex formation, the treatment produced a reduction in the target itself, with a strong reduction of DKK-1 protein levels in a concentration-dependent manner in both cell lines, from 0.2 μM onward (Figure 3B). Similar to our previous results with genetic inhibition, WAY-262611 treatment produced a significant reduction in cell proliferation (Figure 3C). In addition, cell invasiveness was also reduced with the treatment (Figure 3D).

### 2.5. WAY-262611 Treatment Produced an Increase in β-Catenin Levels, an Induction of Myogenic Markers and a Decrease in FAK Levels

Regarding β-catenin, both protein levels and promoter activity (measured by luciferase activity) were significantly increased by WAY-262611 in both cell lines (Figure 3E,F). However, in concordance with our previous observations with DKK-1 genetic inhibition, the levels of c-Myc were not affected by the treatment. Additionally, in agreement with the results obtained with genetic DKK-1 inhibition, pharmacological inhibition produced an induction of MYOD1 and myogenin in both cell lines (Figure 3F). Importantly, FAK levels were also reduced after treatment in both cell lines (Figure 3F).

### 2.6. WAY-262611 Treatment Decreased the Survival of Intravenously Injected Tumor Cells

**Figure 4:**
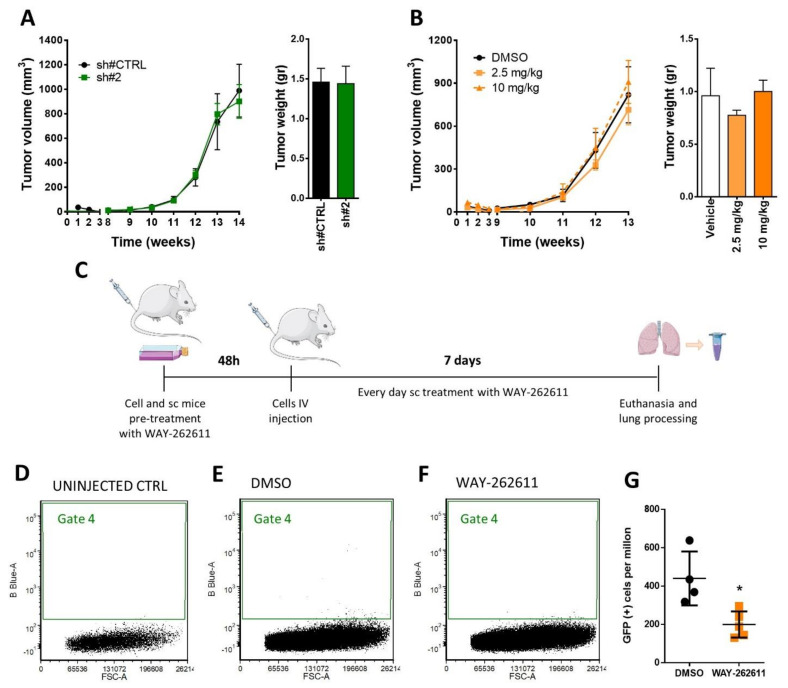
Analysis of DKK-1 inhibition by genetic knockdown and WAY-2626211 in mouse models. Orthotopic models were established by intramuscular injection of RD cells. (**A**) Tumor growth kinetics (**left** panel) and tumor weight at the end of the experiment (right panel) were evaluated during 14 weeks in mice injected with RD cells carrying the control plasmid or the sh#2. (**B**) Mice injected with RD wt cells were treated with vehicle and two different doses of WAY-262611. The tumor volume was determined weekly during the 13 weeks of treatment (**left** panel). At the end of the experiment, the tumor weight was also measured (**right** panel). (**C**) Experimental design corresponding to the experimental metastasis model. After 7 days of treatment, whole lungs were extracted and, after tissue homogenization, samples were analyzed by flow cytometry to identify GFP + cells, detected in Gate 4. (**D**) In mice without tumor cell injection, no GFP + cells were detected in the lungs (0.00%). Representative plots derived from (**E**) cytometry analysis of control and (**F**) treated mice are also shown. (**G**) Final data were plotted as GFP + cells/million events in both control and treated mice. * *p* < 0.05.

In order to analyze the effects of WAY-262611 treatment on tumor growth in vivo, we established an orthotopic RMS mouse model and tested the antitumor activity of both genetic and pharmacological inhibition (with WAY-262611 at two concentrations). Neither the tumor volume nor tumor weight were affected by the genetic inhibition nor treatment (Figure 4A,B). Owing to the reduction of invasion observed in vitro with WAY-262611, we next analyzed the possible role of DKK-1 inhibition in early metastasis steps in vivo, specifically the viability of tumor cells in lung tissue after the tail-vein injection was measured. For this purpose, mice were intravenously injected with GFP-transduced RD cells and the number of viable GFP-positive cells detected in lungs were analyzed at 7 days post-injection (Figure 4C). With this technique it is possible to detect and quantify the small number of cells that reach the lung tissue. The number of viable GFP-positive cells detected in the lung after 7 days of treatment (Gate 4) showed a clear decrease in mice subcutaneously treated with WAY-262611 (Figure 4E,F). The quantification of these cells showed a statistically significant drop from a mean of 440 GFP-positive cells/million for control cells versus 200 GFP-positive cells/million for WAY-262611 treatment (Figure 4G). Since no anti-proliferative effects were observed with genetic inhibition and WAY-262611 in vivo (Figure 4A,B), we concluded that the reduction in GFP-positive cell detection was due to an impaired survival of cells that were intravenously injected.

## 3. Discussion

The Wnt/β-catenin signaling pathway has been described as an important player in tumorigenesis in several malignancies [1]. Its regulation involves different proteins commonly referred to as Wnt antagonists, which encompass several protein families [2,3]. Among them, DKK-1 is the most studied member of DKK family, and has been shown to have therapeutic potential in different diseases [4]. Although there are several compounds able to inhibit DKK-1 (such as WAY-262611 [5]), these molecules have not been tested in RMS so far.

In our gene expression analyses, we observed an upregulation of different members of the DKK family in RMS, both in patients and in cultured cell lines. Presumably, the overall high levels of DKK family components in RMS would entail an inactivation of the Wnt/β-catenin signaling pathway. These findings are in accordance with previous studies that reported a low expression of β-catenin in RMS, evidencing an inactive state of Wnt/β-catenin signaling [15,16]. We hypothesize that this inactivation could be explained, at least in part, by the high levels of several Wnt antagonists, such as DKK-1, DKK-3 and/or DKK-4. DKK-1 in particular has been found to be overexpressed in a plethora of tumors with a clear oncogenic function [17,18,19]. In this context, it has been demonstrated that the reactivation of the Wnt/β-catenin pathway by Glycogen synthase kinase 3 inhibitors has anti-oncogenic effects in RMS [20]. Moreover, other studies have also shown that Wnt/β-catenin reactivation is able to produce antitumor effects in melanoma [21] and breast cancer [22].

In our work on RMS, the observed increase in β-catenin expression and the promotion of its nuclear location, both observed after genetic or pharmacological DKK-1 inhibition, did not produce significant variations in the canonical target c-Myc. This result is in agreement with previous observations that point out a relevance of the non-canonical Wnt signaling pathway in RMS [23,24]. However, the consistent activation of myogenic markers such as MYOD1 and myogenin, together with the reduction of FAK expression, suggests the triggering of a coordinated anti-oncogenic response. This idea is supported by a previous study, which described that the activation of Wnt/β-catenin signaling would trigger an anti-oncogenic role in RMS [20]. Concordantly, in our work, DKK-1 inhibition promoted the increase of muscle differentiation markers in vitro, such as myogenin and MYOD1. These findings support, as previously reported, a relationship between β-catenin, myogenin and MYOD1 [9,25]. Thus, we hypothesize that the activation of the Wnt pathway after DKK-1 chemical inhibition would promote the interaction between β-catenin and myogenin and MYOD1, probably facilitating differentiation, which may lead to a reduction in cell proliferation, as well as cell invasion. This hypothesis has been also previously described in RMS, where the decrease of DKK-1 expression induced by the inhibition of EHMT2 (a lysine methyltransferase) produced the differentiation of ERMS cells together with a decrease in proliferation [9]. In this work, the DKK-1 genetic inhibition also led to an increase in myogenic markers in RMS cell lines, concurring with our findings. In fact, the induction of cell differentiation is a well-described strategy that has shown promising results in preclinical models in a variety of neoplastic diseases, such as acute leukemia or ovarian cancer [26,27]. Our results confirm the activation of the expression of myogenic markers after DKK-1 genetic and pharmacological inhibition. Despite morphological changes were observed in RD cell line after DKK-1 inhibition, we cannot conclude that differentiation was induced in all cell lines analyzed, since in the CW9019 cell line no changes in morphology were observed (data not shown).

In addition to the induction of myogenic markers, the consistent FAK downregulation observed after genetic DKK-1 inhibition or WAY-262611 treatment may also contribute to the observed anti-oncogenic effects. In support of this observation, a previous work showed that treatment with WAY-262611 was able to inhibit the expression of FAK in a rheumatoid arthritis model [4]. FAK is a well-known pivotal regulator of different oncogenic processes, such as migration, invasion and cell survival, among others [28,29], and its relationship with Wnt signaling is well documented; however, whether FAK is an upstream or a downstream regulator of Wnt signaling is a matter of controversy and appears to be strongly context specific [29]. Our findings indicated that FAK is downregulated by the activation of the Wnt/β-catenin pathway, as described in human mesothelioma cells and in prostate cancer models, where the inactivation of Wnt produced the activation of FAK [29,30]. Moreover, the upregulation of FAK has been described as a negative regulator of myogenesis [31], facilitating the activation of the cell cycle and playing a negative role in myoblast differentiation [32]. Thus, there is prior evidence that makes a direct relationship in our RMS models plausible, between Wnt/β-catenin activation and FAK downregulation and, in turn, between FAK downregulation and the increased expression of MYOD1 and myogenin.

Despite the effects on proliferation and myogenic markers observed in vitro, genetic inhibition or treatment with WAY-262611 did not produce significant effects on tumor growth in the primary mouse models. These results coincide with a previous work where they show that reactivation of the β-catenin pathway did not produce significant effects on proliferation in a RMS in vivo model [23]. Indeed, it has been described that activation of Wnt/β-catenin signaling produces differentiation of RMS cells without a clear effect in proliferation and/or apoptosis [24]. Regarding the results of the primary models, two possible scenarios could explain this discrepancy. On the one hand, the stromal DKK expression in mice could affect the tumor inhibition produced by WAY-26211. For instance, it has been shown that DKK-3, produced by benign stroma, increases the tumorigenicity of prostate cancer cells [33]. Furthermore, in the same work, the authors showed contrasting effects of DKK-3 inhibition in prostate stromal and epithelial cells, suggesting that extracellular factors can strongly influence prostate carcinogenesis. In our case, we can hypothesize that stromal proteins secreted from the surrounding skeletal muscle could compensate the DKK-1 inhibition exerted by WAY-262611. On the other hand, given the functional redundancy of the four DKK family components, we hypothesized that it is also highly plausible that the presence of other members of the family could counteract the inhibition of DKK-1. Besides the DKK family, there are other Wnt/β-catenin signaling antagonists [34], and several overlapping functions in different cellular processes have been reported [35]. Usually, each Wnt antagonist is studied separately, but the relationship with other members of the same family, or even proteins belonging to other families, cannot be overlooked, since altogether they constitute an intricate network that regulates and modulates the activity of the pathway. Therefore, a deeper understanding of the whole process of extracellular Wnt/β-catenin regulation—including DKKs, SFRPs, WIF1, Cerberus, Tiki1, APCDD1, SOST, Wise, MESD, Waif, IGFBP-4 and Shisa, among others—would be necessary to improve strategies based on Wnt/β-catenin activation as a therapeutic option in RMS. Thus, the plethora of possibilities for Wnt pathway modulation make it very difficult to elucidate the exact mechanism of regulation, and probably may explain the differences observed between our in vivo and vitro models, thereby limiting the translationality of the inhibition of a single protein, such as DKK-1.

Despite the lack of activity of the compound WAY-262611 in mouse primary RMS tumors, its notable effects on FAK expression and in cell invasiveness observed in vitro led us to hypothesize a possible role of DKK-1 in RMS metastasis process. With the aim of testing this hypothesis, we analyzed the effect of DKK-1 inhibition produced by the compound WAY-262611 in an experimental mouse model of metastasis, by which it is possible to quantify the cells that reach the lung. Our results suggest that DKK-1 inhibition reduce the number of tumor cells in lungs, thereby evidencing an essential role for DKK-1 in this early metastasis step, which would not be completely compensated—in contrast to the results in the primary tumor model—by other DKK proteins and/or other Wnt antagonist proteins. In fact, the relationship between DKK-1 and metastasis has been previously described [36,37]. In the work of Malladi et al. [36], a direct role for DKK-1 in the metastatic process in breast and lung cancers was described. Interestingly, in breast cancer, DKK-1 inhibition has contradictory effects depending on the metastatic target tissue. Thus, DKK-1 inhibition impaired lung metastasis, in agreement with our results, but promoted bone metastasis [38]. These observations highlighted the importance of the cellular origin and tissue microenvironment for DKK-1 functionality in each particular type of tumor.

## 4. Materials and Methods

### 4.1. Database and mRNA Expression Analyses

Analysis of gene expression data from patients was performed on publicly available data from the R2: Genomic Analysis and Visualization Platform (http://r2.amc.nl, accessed on 15 December 2020). Muscle tissue was considered as the “normal” counterpart. For this latter group, data from two datasets were used: “Normal Muscle—Gordon—22—MAS5.0—u133p2” and “Normal Muscle—Hofman—121—MAS5.0—u133a”. Only data from control donors were taken into account for the analysis. For the Gordon database, only the young subset of patients was included to avoid age-related bias. The “Tumor Rhabdomyosarcoma—Davicioni—147—MAS5.0—u133a” set was used for RMS patients.

Raw expression data from RMS cell lines were obtained from the public database GEO2R (https://www.ncbi.nlm.nih.gov/geo/geo2r/, accessed on 13 January 2021) (Accession number: GSE8840 [14]). Raw data were normalized and analyzed by AltAnalyze software (http://www.altanalyze.org/, accessed on 24 February 2021) in order to obtain the relative expression values for each DKK gene in each cell line.

### 4.2. Cell Culture

All RMS cell lines were grown in MEM media (Biowest, Riverside, MO, USA). Culture media were supplemented with 10% fetal bovine serum (Sigma-Aldrich, Saint louis, MO, USA), 2 mM L-glutamine, 1 mM sodium pyruvate, 1× non-essential amino acids, 100 U/mL penicillin and 0.1 mg/mL streptomycin (all from Biowest). Cell lines were cultured at 37 °C in a 5% CO_2_ with humidified atmosphere. Cell lines were purchased from the American Type Culture Collection, except CW9019, which was kindly provided by Dr. Jacklyn Biegel’s laboratory.

### 4.3. Lentiviral Transduction

Genetic knockdown of DKK-1 was carried out by shRNA technology using the lentiviral vector pGIPZ (GE Healthcare Dharmacon, Lafayette, CO, USA). Lentiviral particles were generated in HEK293T as previously described [39]. Briefly, 2 × 10^5^ cells (for RD and CW9019) were seeded in 60 mm dishes and incubated overnight. Then, 48 h after infection, selection with puromycin (1 μg/mL, Sigma-Aldrich, Saint louis, MO, USA) was performed to select positive-transduced cells. The knockdown efficiency was tested by Western blot (WB). The selected DKK-1 shRNAs were clones V2LHS_19944 (for sh#1) and V3LHS_412074 (for sh#2) (both from Dharmacon, Lafayette, CO, USA).

### 4.4. Cell Proliferation Assay and IC50 Determination

For the proliferation assay, cells were seeded in 6-well plates at a density of 3 × 10^4^ cells/well for RD, and at 2.5 × 10^4^ cells/well for CW9019. At different time points (3, 5 and 7 days), cells were trypsinized and resuspended in coulter Isoton II diluent (Beckman Coulter). To evaluate the number of cells on each time point, cell suspensions were analyzed in a Z series Coulter counter (Beckman Coulter, Brea, CA, USA) following the manufacturer’s instructions. Each condition was analyzed in triplicate in three independent assays.

For IC50 determination, cells were seeded in 96-well plates (2 × 10^3^ cells/well for RD and 6 × 10^3^ for CW9019). The following day, WAY-262611 was added at different concentrations. After 72 h, cells were dyed with 0.5% crystal violet (Sigma-Aldrich, Saint louis, MO, USA). After overnight drying, crystals were dissolved with 15% acetic acid solution and absorbance was quantified at 590 nm with an Epoch Microplate Spectrophotometer (Biotek, Winuschi, VT, USA). Absorbance data were used to calculate the percentage survival under each condition. Three independent assays with six replicates per condition were performed.

### 4.5. Transwell Cell Invasion Assay

Invasion assay was performed as previously described in detail [40]. Briefly, 1 × 10^5^ RD or CW9019 cells were seeded in an 8 μm pore size transwell (Corning), previously coated with 25 µL of BD Matrigel™ (Corning). After 24 h of incubation, migrated cells were washed, fixed and stained with Hoechst-33342 (5 ng/mL). Cells were counted under the fluorescent microscope. For each condition, three wells from three independent assays were analyzed.

### 4.6. Western Blot

Proteins were extracted using RIPA buffer (Thermo Fisher Scientific, Waltham, MA, USA), and the protein content was measured using the DC Protein Assay Kit (BioRad, Hercules, CA, USA). Then, 20–40 μg of total protein were resolved by SDS-PAGE electrophoresis and transferred onto a PVDF membrane. After blocking, membranes were incubated with the following primary antibodies: Anti-DKK-1 (diluted 1:250, cell signaling, Danvers, MA, USA, 4687S), Anti-c-Myc (diluted 1:100, Santa Cruz Biotechnology, Franklin Lakes, NJ, USA, sc-40), Anti-MYOD1 (diluted 1:1000, Abcam, Cambridge, UK, ab16148), Anti-Myogenin (diluted 1:250, BD Pharmingen, Franklin Lakes, NJ, USA, 556358), Anti-FAK (diluted 1:1000, cell signaling, Danvers, MA, USA, 3285S), Anti-β-catenin (diluted 1:1000, BD Bioscience, Franklin Lakes, NJ, USA, 610154), Anti-Lamin A (diluted 1:1000, Santa Cruz Biotechnology, Franklin Lakes, NJ, USA, sc-20681) and Anti-Actin (diluted 1:10,000, Santa Cruz Biotechnology, Franklin Lakes, NJ, USA, sc-1616). Anti-goat (diluted 1:2000, Dako, Glostrup, Denmark, P0449), anti-mouse (diluted 1:2000, Dako, Glostrup, Denmark, P0260) and anti-rabbit (diluted 1:5000, Sigma, Saint louis, MO, USA, A0545) were used as secondary antibodies. Uncropped membranes and values of intensity ratio for each band are shown in Supplementary Figure S1.

### 4.7. Luciferase Assay of β-Catenin Transcriptional Activity

To analyze the activation of Wnt/β-catenin signaling, growing RD and CW9019 cells were co-transfected with M50 Super 8x TOPFlash plasmid (Addgene, Watertown, MA, USA, 12456) or M51 Super 8x FOPFlash plasmid (Addgene, Watertown, MA, USA, 12457) together with Renilla reporter plasmid pRL-TK (Promega, Madison, Wisconsin), using X-tremeGENE 9 DNA Transfection Reagent (Roche, Basel, Switzerland), following the manufacturer’s instructions. After 24 h, cells were harvested and a luciferase assay was performed using the Dual-Glo Luciferase Assay System (Promega, Madison, Wisconsin) following the manufacturer’s instructions. Luminescence was measured using the Appliskan reader (Thermo Fisher Scientific, Waltham, MA, USA). The luciferase activity of each condition was normalized for transfection efficiency to its Renilla luciferase activity.

### 4.8. Primary Tumor and Metastasis Models in Mice

For the primary orthotopic tumor model, 1 × 10^6^ RD cells were injected into the right gastrocnemius muscle of SCID mice (Charles River Laboratories, Wilmington, MA, USA). All mice were maintained under pathogen-free conditions. To analyze the effects of DKK-1 genetic inhibition on tumor growth in vivo, mice were injected with RD cells carrying the control plasmid or infected with sh#2. For the pharmacological inhibition, mice injected with RD wt cell line were randomized into 3 groups and treated daily with subcutaneous vehicle solution, 2.5 mg/kg or 10 mg/kg of the WAY-262611 compound. Limb volume was measured with a caliper to calculate the tumor volume using the formula$\;V=\frac{3}{4}\pi \times {\left(\frac{\left(H\;+\;A\right)}{4}\right)}^{3}$. Tumor size (>1 cm in diameter in any dimension), acute weight loss (>10% of total body weight) or poor general appearance of the animal were used as ethical endpoint criteria.

For the lung metastasis model, 3 × 10^6^ RD cells previously transduced with a GFP reporter vector pGIPZ (control plasmid) were intravenously injected through the tail vein of SCID-beige mice. Prior to injection, RD cells were pre-treated in vitro with DMSO (vehicle, control group) or with WAY-262611 (0.2 µM) (treated group) for 48 h. After cell injection, mice were treated daily with subcutaneous injection (for 7 days) with the vehicle (15% DMSO in NaCl Meinsol, Fresenius Kabi, Bad Homburg, Germany) (control group) or with 10 mg/kg of WAY-262611 in the same solvent (treated group). At the end of the experiment, the lungs were enzymatically dissociated with collagenase A (Sigma, Saint louis, MO, USA) and analyzed by FACS (FacsAria, BD Bioscience, Franklin Lakes, NJ, USA). Analyses were carried out as previously described [40]. All procedures were approved by the Ethics Committee of Animal Experimentation of the Vall d’Hebron Research Institute (CEEA 30/14) and were in line with EU directive 2010/63/EU.

### 4.9. Statistical Analysis

Statistical analyses were performed using GraphPad Prism Software (version 6.01). At least three independent replicates were performed for each experiment. The mean ± SD was graphed, and statistical significance was determined by Student’s *t*-test or ANOVA, considering * *p* < 0.05, ** *p* < 0.01, *** *p* < 0.001 and **** *p* < 0.0001. Bonferroni correction was applied for multiple comparisons when more than two datasets were analyzed.

## 5. Conclusions

In this work, we provide an initial characterization of the DKK family in RMS with a special emphasis on DKK-1. However, the possible role of other DKK members in RMS needs to be further investigated. Our results indicated that DKK-1 inhibition produces anti-oncogenic effects in RMS, both in vitro and in vivo. Furthermore, our data suggest a possible regulatory link between reactivation of Wnt/β-catenin signaling—promoted by DKK-1 inhibition—and the differentiation of RMS cells via FAK downregulation and a concomitant promotion of MYOD1 and myogenin. Therefore, we identify DKK-1 as a pro-oncogenic protein in RMS and as a putative molecular target, which may have potential in the treatment of this soft tissue sarcoma. The most remarkable effect observed by DKK-1 pharmacological inhibition was the impairment of the survival of tumor cells in lungs, while primary tumor growth was not affected by genetic inhibition or WAY-262611 treatment. Our work suggests that DKK-1′s specific role is essential for early metastasis steps; however, the fact that tumor growth is not affected by DKK-1 inhibition makes us think that the most plausible hypothesis is that other DKKs, or even Wnt antagonists belonging to other families, may compensate the lack of DKK-1 function. Therefore, to completely unravel the molecular mechanisms involved, more studies are needed. These studies should take into account the intricate regulation of the pathway to permit the implementation of a therapy based on the reactivation of Wnt/β-catenin signaling.

## Supplementary Materials

The following are available online at https://www.mdpi.com/article/10.3390/ijms222312921/s1.

## Abbreviations

RMS	rhabdomyosarcoma
DKK	Dickkopf
LRP	low-density lipoprotein receptor-related protein
Wnt	Wingless-INT
Fz	Frizzled
FAK	focal adhesion kinase
MYOD1	myogenic differentiation 1
WB	Western blot
