# Supplementary material for: Dickkopf-1 Inhibition Reactivates Wnt/β-Catenin Signaling in Rhabdomyosarcoma, Induces Myogenic Markers In Vitro and Impairs Tumor Cell Survival In Vivo
Source: Int J Mol Sci. 2021 Nov 29;22(23):12921. doi: 10.3390/ijms222312921 (PMC8657544; doi:10.3390/ijms222312921)

FIGURE S1. Uncropped western blot figures.

Uncropped western blot Figure 1C

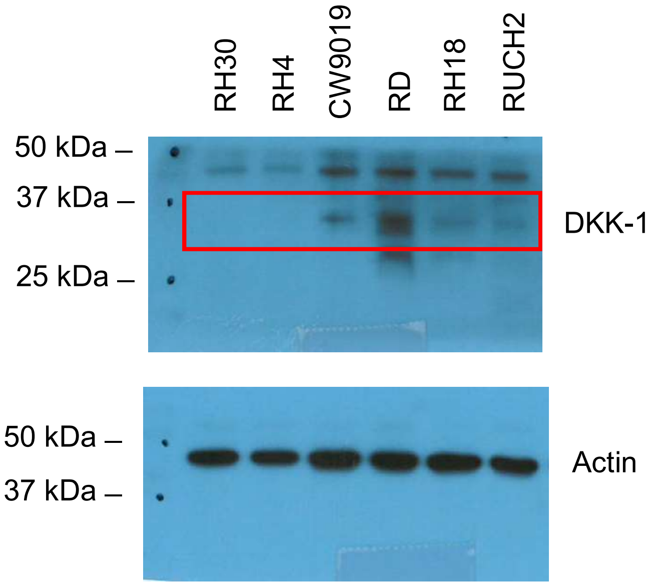

NOTE. Actin was developed after stripping of DKK-1 detection

WB quantification for DKK-1: intensity for each band was measured with ImageJ software. DKK-1 was normalize to actin in order to obtain a relative expression ratio:

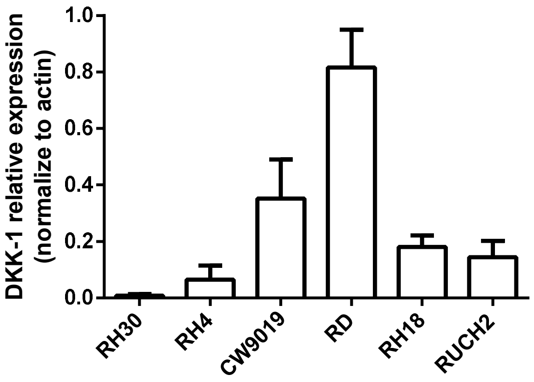

Uncropped western blot Figure 2A

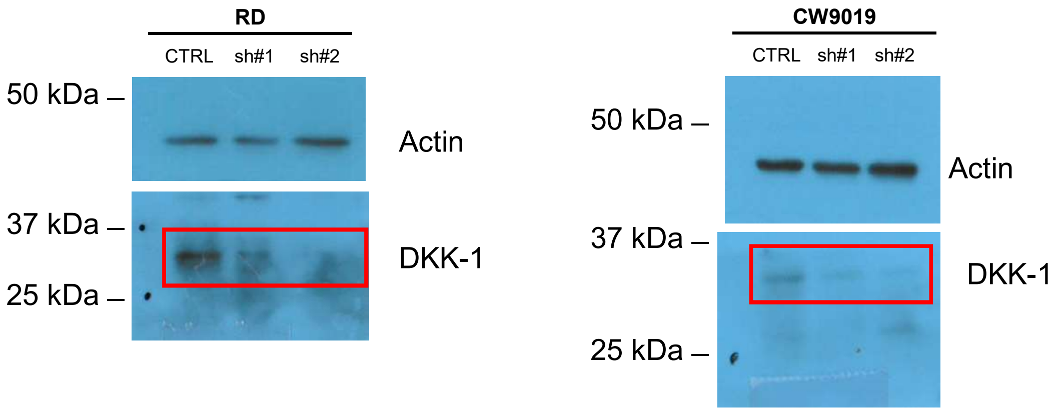

WB quantification for DKK-1: intensity for each band was measured with ImageJ software. DKK-1 intensity was normalize to actin. Since a control was included in the experimental design, values were then referred to the corresponding control (=1) in order to obtain a relative expression ratio:

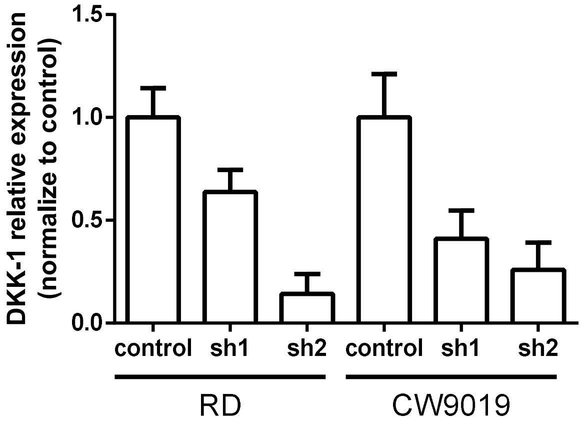

FIGURE S1. Uncropped western blot figures (cont.).

Uncropped western blot Figure 2C

Complete cell lysate

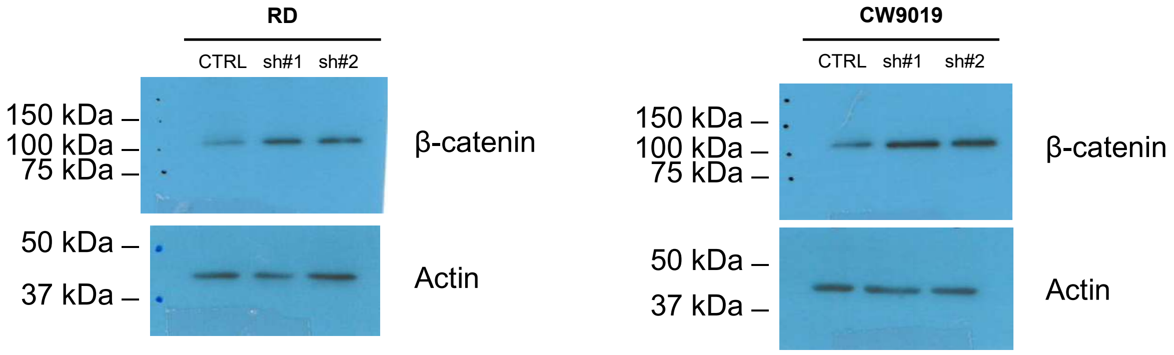

Nuclear fraction

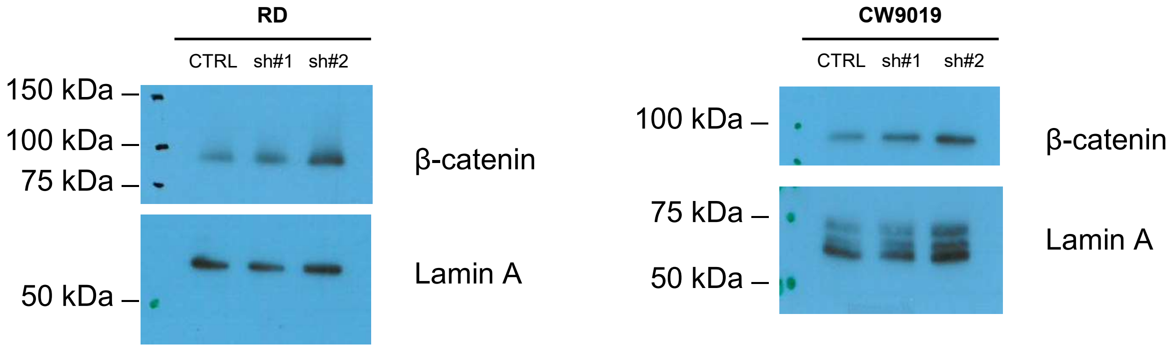

WB quantification for B-catenin: intensity for each band was measured with ImageJ software. B-catenin intensity was normalize to actin for the complete cell lysate, and to Lamin A for nuclear fraction. Since a control was included in the experimental design, values were then referred to the corresponding control (=1) in order to obtain a relative expression ratio:

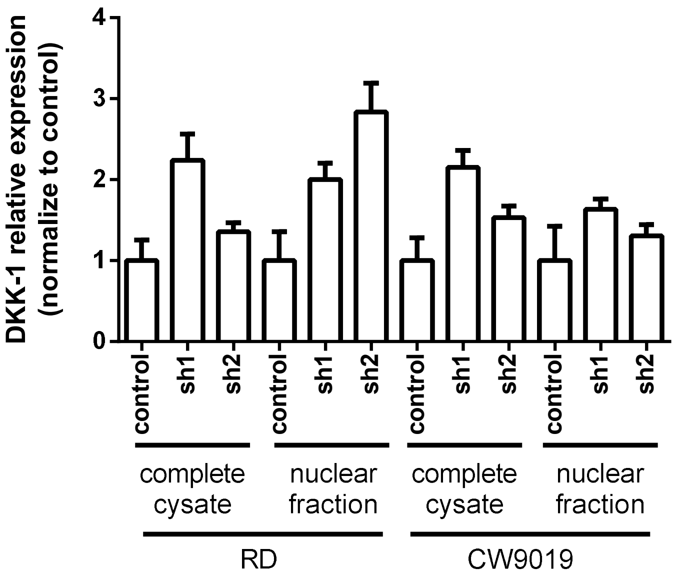

FIGURE S1. Uncropped western blot figures (cont.).

Uncropped western blot Figure 2E

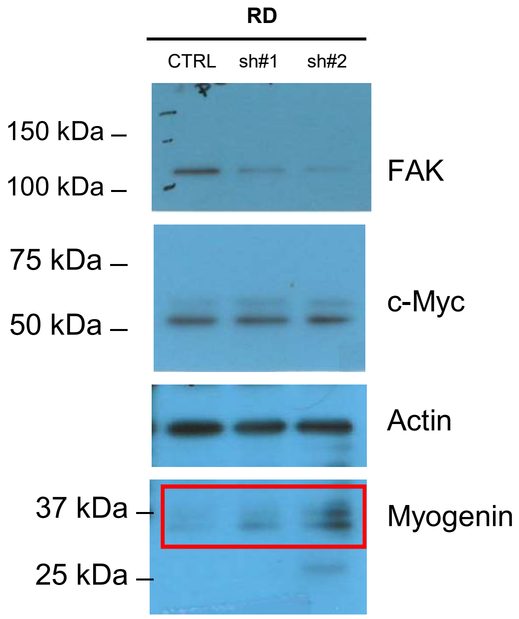

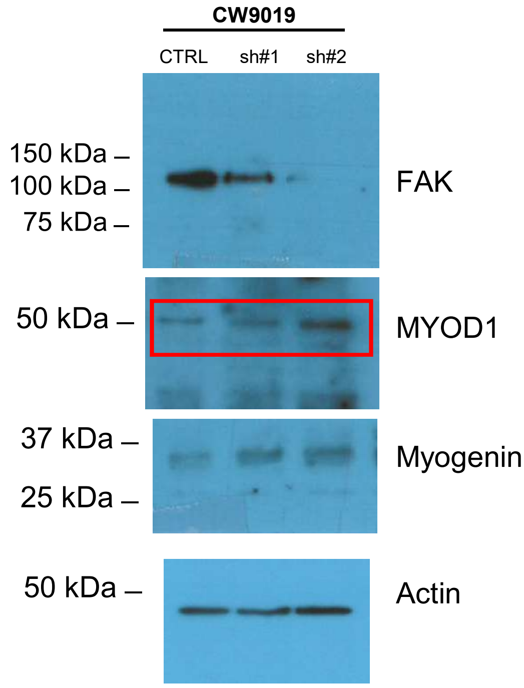

NOTE: For this blot, actin was developed after stripping of MYOD1 membrane

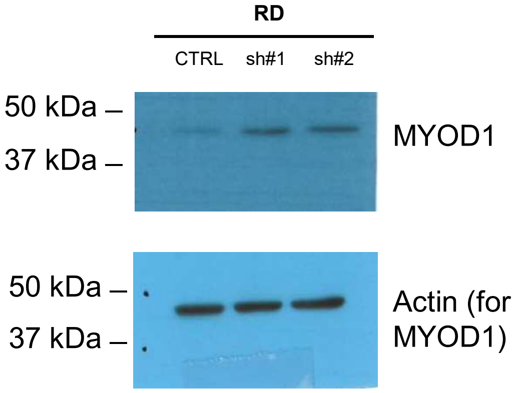

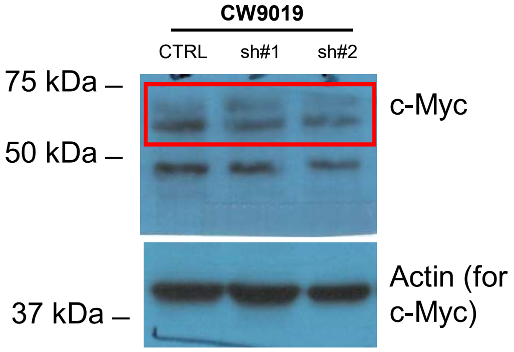

NOTE: For this blot, actin was developed after stripping of MYOD1 membrane

WB quantification: intensity for each band was measured with ImageJ software. Intensity for each protein was normalize to the corresponding actin. Since a control was included in the experimental design, values were then referred to the corresponding control (=1) in order to obtain a relative expression ratio:

RD cell line

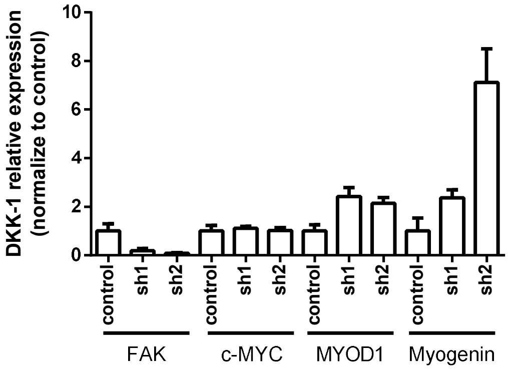

CW9019 cell line

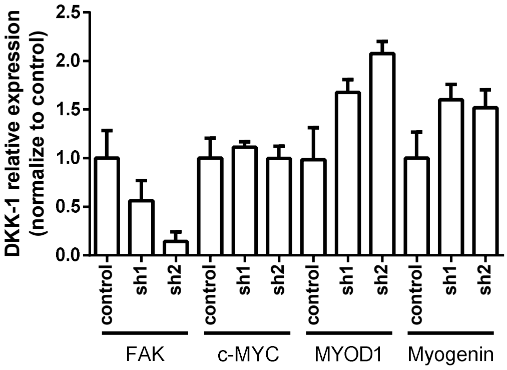

FIGURE S1. Uncropped western blot figures (cont.).

Uncropped western blot Figure 3B

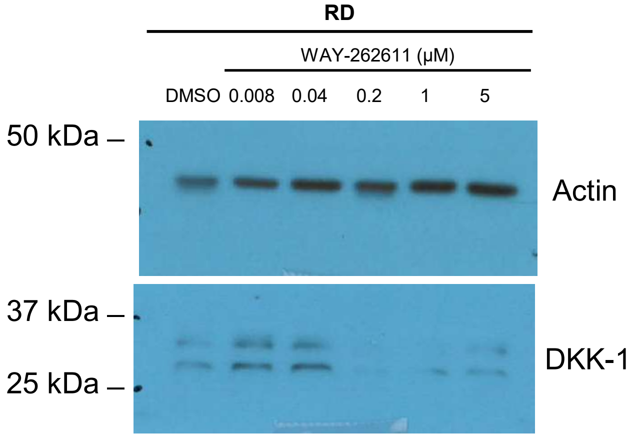

WB quantification for DKK-1: intensity for each band was measured with ImageJ software. DKK-1 intensity was normalize to actin. Since a control (DMSO) was included in the experimental design, values were then referred to the corresponding control (=1) in order to obtain a relative expression ratio:

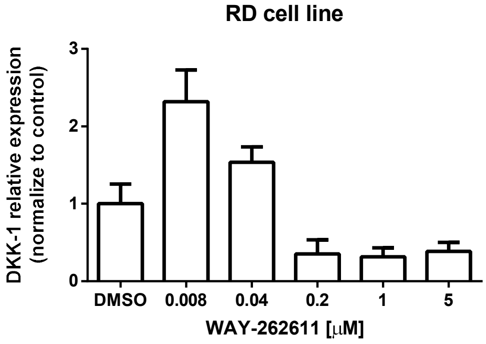

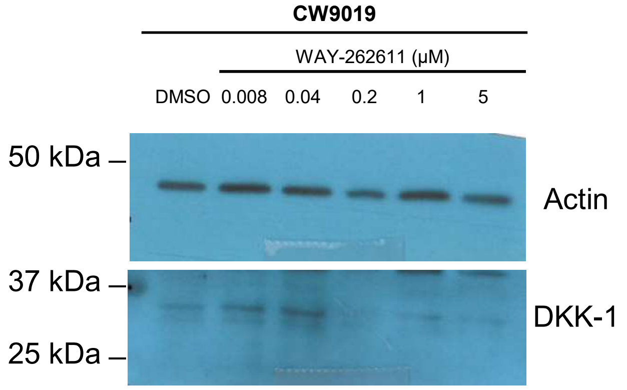

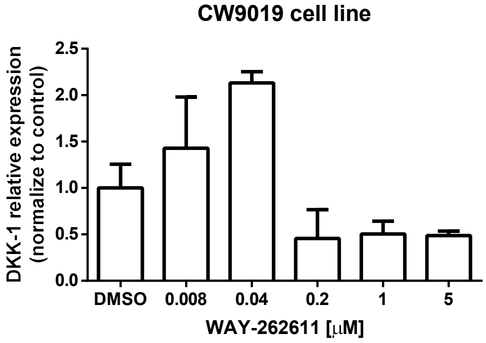

FIGURE S1. Uncropped western blot figures (cont.).

Uncropped western blot Figure 3F

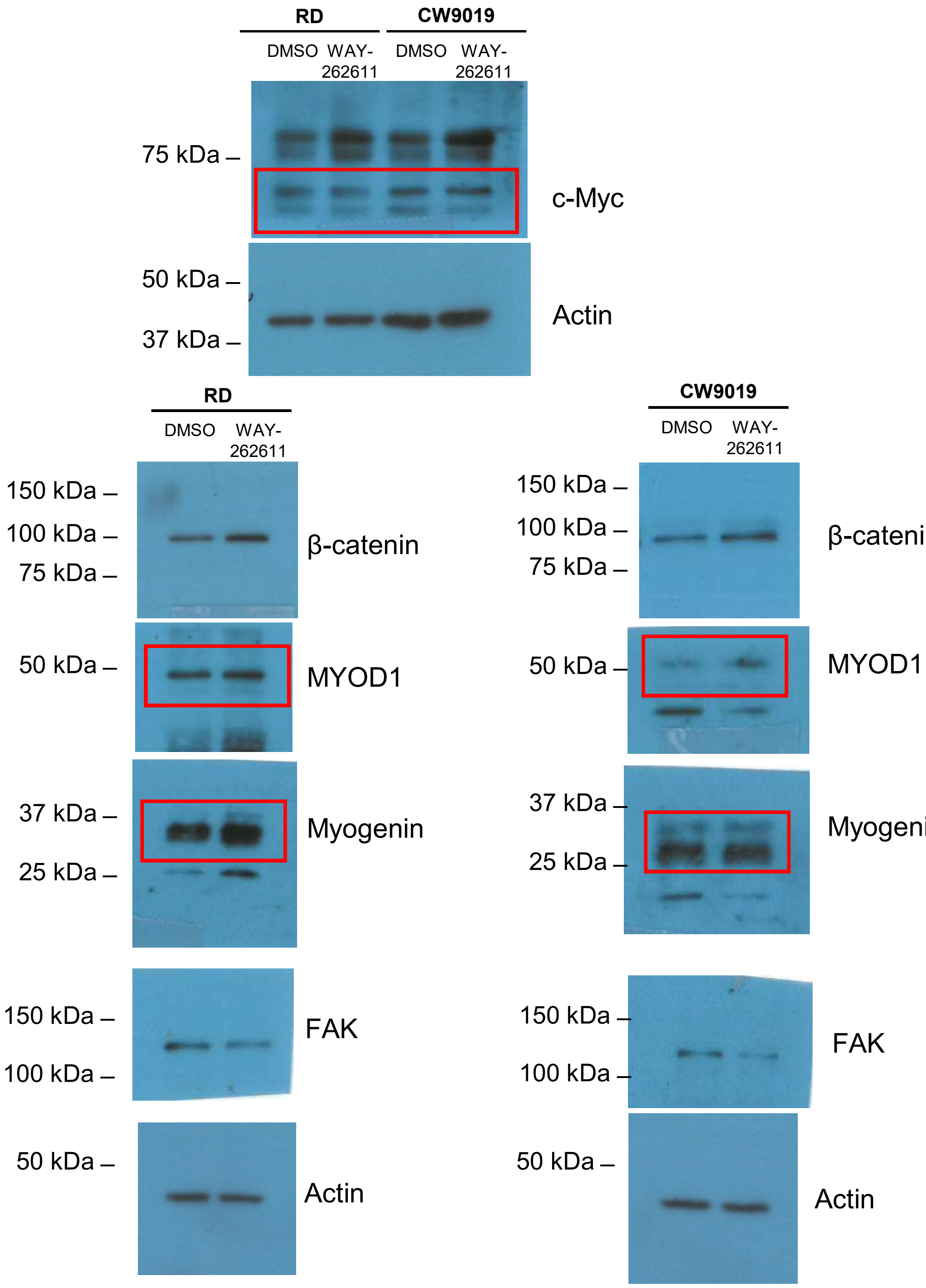

NOTE: FAK was developed after stripping of β-catenin membrane

Actin was developed after stripping of MyoD1 membrane

WB quantification: intensity for each band was measured with ImageJ software. y for each protein was normalize to the corresponding actin. Since a control (DMSO) was included in the experimental design, values were then referred to the corresponding control (=1) in order to obtain a relative expression ratio:

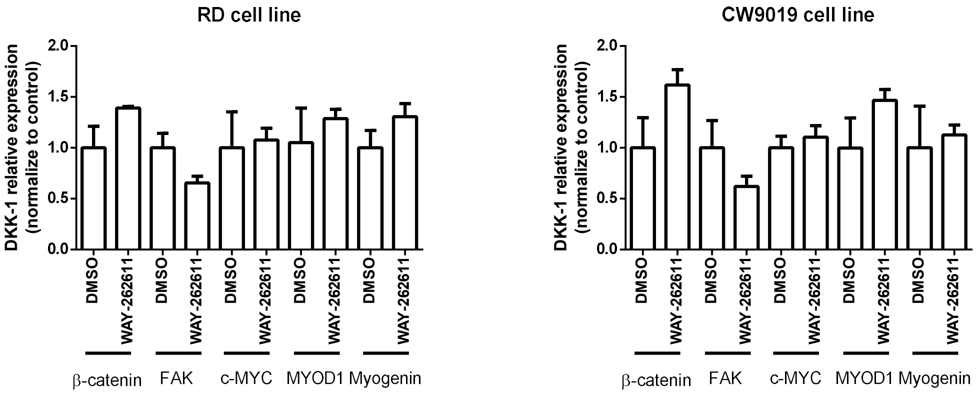

Supplement: Supplementary file 1 [file ijms-22-12921-s001.zip › ijms-1451423-supplementary.pdf]
